# Antibiotic resistance profile of Staphylococcus aureus clinical isolates from Nigeria

**DOI:** 10.1186/2047-2994-4-S1-P195

**Published:** 2015-06-16

**Authors:** OO Ayepola, L Egwari, GI Olasehinde

**Affiliations:** 1 Biological Sciences, Covenant University, Ota, Nigeria

## Introduction

Hospital-acquired infections with *Staphylococcus aureus* have increased over the years and the rise in incidence has been accompanied by a rise in antibiotic-resistant strains notably methicillin-resistant *S. aureus* (MRSA) and more recently vancomycin-resistant strains. In order to have adequate information for treatment of infections caused by *S. aureus*, it is important to understand trends in the antibiotic-resistance patterns as well as diversity of strains across geographical regions.

## Objectives

The aim of this study was to provide information on the antibiotic resistance profile and molecular characteristics of *S. aureus* strains from Nigeria.

## Methods

A total of 209 non-duplicate *S. aureus* isolates obtained from clinical infections in eight medical centres were analyzed. Identification and antimicrobial susceptibility profile was performed with the automated VITEK-2 system. Detection of antibiotic resistance and virulence genes in the *S. aureus* strains was by polymerase chain reaction.

## Results

Resistance was observed against penicillin (97.1%); trimethoprim/sulfamethoxazole (83.7%), tetracycline (13.8%), levofloxacin (5.7%) and gentamicin(4.8%). All strains were susceptible to azithromycin, clarithromycin, erythromycin, clindamycin, linezolid, vancomycin, nitrofurantoin, fusidic acid, mupirocin and rifampicin. The β-lactamase (*blaZ*)gene was found in 95% of allstrains (n=198) while 2.87% (n=6) possessed the *mecA* gene. The staphylococcal cassette chromosome *mec* (SCC *mec*) typing of MRSA strains detected SCC *mec* type IV in one strain. A particular MRSA strain was the only strain found to be resistant to teicoplanin, tigecycline and fosfomycin. Fifty-six percent of the strains possessed the panton valentine leukocidin (PVL) encoding gene.

## Conclusion

A rise in PVL-positive *S. aureus* strains in Africa is of great concern as this could promote the emergence of highly virulent strains. The continuous surveillance of antibiotic resistance in *S. aureus* is important to prevent the spread of multidrug resistant strains.

## Disclosure of interest

None declared.

